# The effects of genomic polymorphisms in one-carbon metabolism pathways on survival of gastric cancer patients received fluorouracil-based adjuvant therapy

**DOI:** 10.1038/srep28019

**Published:** 2016-07-26

**Authors:** Tingting Zhao, Zhi Xu, Dongying Gu, Peng Wu, Xinying Huo, Xiaowei Wei, Yongfei Tang, Weida Gong, Ming-Liang He, Jinfei Chen

**Affiliations:** 1 Department of Oncology, Nanjing First Hospital, Nanjing Medical University, Nanjing, 210006, China; 2 Department of Surgery, Yixing People’s Hospital, Yixing, 214200, China; 3 Department of Surgery, Yixing Cancer Hospital, Yixing, 214200, China; 4 Department of Biomedical Sciences, City University of Hong Kong, Hong Kong; 5 CityU Shenzhen Research Institute, Shenzhen, 518000, China; 6 Jiangsu Key Lab of Cancer Biomarkers, Prevention and Treatment, Collaborative Innovation Center for Cancer Personalized Medicine, Nanjing Medical University, Nanjing, China

## Abstract

5-fluorouracil (5-FU) is widely used to treat patients with gastric cancer (GC). However, the response rate is quite heterogeneous. The single nucleotide polymorphisms (SNPs) and their interactions of genes in the one-carbon metabolism (OCM) pathway, including Methylenetetrahydrofolate reductase (*MTHFR*), Methionine synthase reductase (*MTRR*), Methionine synthase (*MTR*), and Thymidylate synthase (*TS*), significantly affect 5-FU metabolism. In this study, 650 stage II-III patients were recruited from 1998 to 2006. Among them, 251 received 5-FU treatment and other 399 patients were untreated. The Cox regression analysis, log-rank tests and Kaplan–Meier plots were adopted. In the chemotherapy cohort, MTRR 66 GA + GG genotypes decreased death risk, however, the protect effect of MTRR 66 GA + GG disappeared when GC patients simultaneously had MTHFR 677TT + TC or MTR 2756GG + GA genotypes. TS 5′-UTR 2R3R + 3R3R genotypes also prolonged overall survival of patients treated with 5-FU. And this favorable prognosis obviously enhanced when GC patients simultaneously had TS 3′-UTR DD + DI and TS 5′-UTR 2R3R + 3R3R genotypes. Our findings showed that the polymorphisms of MTRR 66 A > G and TS 5′-UTR 3R > 2R may be potential prognostic factors for GC patients receiving 5-FU.

Despite annual decline in death rates of the gastric cancer (GC) over the past decade, it is still the second most common cause of cancer death worldwide^1^. China possesses 47% of new global GC cases, with the highest mortality rate among all malignant cancers^2^. Two thirds of GC patients at early or advanced stage recur after curative resection. So, the first line of treatment is chemotherapy followed by surgery for most GC patients^3^. The combination of surgery with chemotherapy obviously improved 5-year survival rates from 49.6% to 55.3% compared with surgery alone^4^. Although new anticancer drugs have been actively developed, 5-fluorouracil (5-FU) still remains one of the most widely used regimes for the treatment of stage II-III GC patients^5^. However, the response rate is only approximately 25%, even if supplemented by leucovorin (CF), which improves the effects of 5-FU^6^. Single-nucleotide polymorphisms (SNPs) of various genes involved in the one-carbon metabolism pathway may play a pivotal role in the efficacy of 5-FU for stage II-III GC patients. Therefore, it is of great importance to identify new biomarkers that can be used as predictors for 5-FU- based chemotherapy.

One-carbon metabolism (OCM) pathway plays an important role in the processes of DNA methylation and DNA synthesis. The enzymes, including *Methionine synthase reductase* (*MTRR*), *Methionine synthase* (*MTR*), *Methylenetetrahydrofolate reductase* (*MTHFR*), and *Thymidylate synthase* (*TS*), are key components to catalyze S-adenosylmethionine (SAM) synthesis from folate update^7^. The antitumor effect of 5-FU depends on an interaction with key enzymes in OCM pathway^8^. As a pyrimidine analogue, 5-FU is metabolized to 5-fluoro-2′-deoxyuridine 5′-monophosphate (FdUMP), which inhibits thymidylate synthase (TS) by forming a ternary complex involving FdUMP, 5-10-methylene tetrahydrofolate (5,10-MTHF) and TS^9^. TS catalyzes methylation of dUMP to dTMP and this is an important process in DNA synthesis^10^. This ternary complex inhibits TS activity and suppresses DNA synthesis, which is thought to be the main mechanism of the antitumor effect of 5-FU^8^. The stabilization and formation of this complex relays on the concentration of 5,10-MTHF. Methylenetetrahydrofolate reductase (MTHFR) catalyzes an irreversible conversion of 5,10-MTHF to 5-methyltetrahydrofolate(5-MTHF)^11^. Reduced enzyme activity of MTHFR lead to an increased concentration of 5,10-MTHF and augment the sensitivity of 5-FU^12^. Until now, 5-FU based adjuvant chemotherapy is widely considered to be the traditional treatment regimen for GC, leading to improvement of quality of life, overall survival (OS) and progression free survival (PFS)^13^. The 5-FU-based regimen normally combines 5-FU with cisplatin (DDP) or oxaliplatin (L-OHP).

The polymorphisms of OCM associated genes may be crucial predictive factors for the GC patients treatment with 5-FU. Two polymorphisms have been identified in TS, including two or three 28-bp repeated (2R/3R) sequences in the 5′-untranslated region (UTR) and a 6-bp insertion/deletion (I/D) in the 3′-UTR^14^,^15^. The 3R allele could enhance TS expression when compared with the 2R allele, and the TS 3′-UTR del6 allele is associated with decreased TS mRNA stability and lower TS expression in comparison with the ins6 allele^15^,^16^. The SNPs of TS influence the response rate to 5-FU by regulating TS expression levels. The MTHFR 677 C > T (rs1801133, Ala222Val) and 1298 A > C (rs1801131, Glu429Ala) polymorphisms are common gene variants that decrease MTHFR activity^17^,^18^. Reduced MTHFR activity increases the accumulation of 5,10-MTHF, which may increase the cytotoxic effect of 5-FU by stabilization of the ternary complex with TS and FdUMP^9^.

Methionine synthase (MTR) catalyzes the transfer of the methyl base from 5-methyl-tetrahydrofolate (5-MTHF) to homocysteine, producing methionine and THF. The function of methionine synthase reductase (MTRR) is to maintain MTR activity^19^. The SNP of MTR 2756 A > G, (rs1805087, Asp868Gly) and MTRR 66A > G, (rs1801394, ILe22Met) may decrease methionine and elevate 5-MTHF levels, thereby increasing the concentrations of 5,10-MTHF^20^. Therefore a decreased activity of the MTR and MTRR would improve the treatment effect of 5–FU-based chemotherapy.

Patients with stage II-III GC are the major group to be dedicatedly cared for anticancer therapy in clinic. Although the effects of SNPs of individual gene or combinations of two genes on the survival benefited from 5-FU have been reported, the synergistic effects of the OCM pathway associated genes polymorphisms on stage II-III GC patients have not been investigated. Based on large-number clinical data analysis, our study systematically investigated the individual and synergistic effects of polymorphisms of TS, MTHFR, MTR and MTR on the prognosis of stage II-III GC patients accepted 5-FU-based adjuvant chemotherapy.

## Results

### Clinical features of the study patients

The clinical features of 650 enrolled patients were summarized in Table 1. The median age was 61.0 years (range, 28–83 years) and 328 of them died during the follow-up of 119.0 months. 251 patients underwent 5-FU-based adjuvant chemotherapy and 399 of them did not receive chemotherapy after surgical resections. In the no adjuvant chemotherapy cohort, the survival time was significantly related to varieties in tumor differentiation, lymph node metastasis and TNM stage (all log-rank p < 0.05). In particular, no chemotherapy patients with III level of TNM stage (MST, 36 months) had a 80.9% notably higher risk of death (HR = 1.809, 95% CI = 1.322–2.477, p < 0.001) when compared with those with II level (MST, 71 months). Patients with lymph node metastasis (MST, 38 months) had a 50.0% higher risk of death (HR = 1.500, 95% CI = 1.056–2.129, p = 0.021) when compared to those without lymph node metastasis (MST, 67 months). Meanwhile, patients with well differentiation (MST, 74months) had 43.6% and 67.5% lower risk of death when compared to those with poorly differentiation (MST, 38 months; HR = 1.436, 95% CI = 1.037–1.989) and mucinous or signet-ring cell (MST, 35 months; HR = 1.675, 95% CI = 1.064–2.636; p = 0.038), respectively. In contrast, no remarkable higher death risks were founded among the chemotherapy cohort.

### MTRR 66 A > G and TS 5′-UTR 2R > 3R polymorphism prolonged overall survival of GC patients receiving 5-FU-based adjuvant chemotherapy

**Figure 1:**
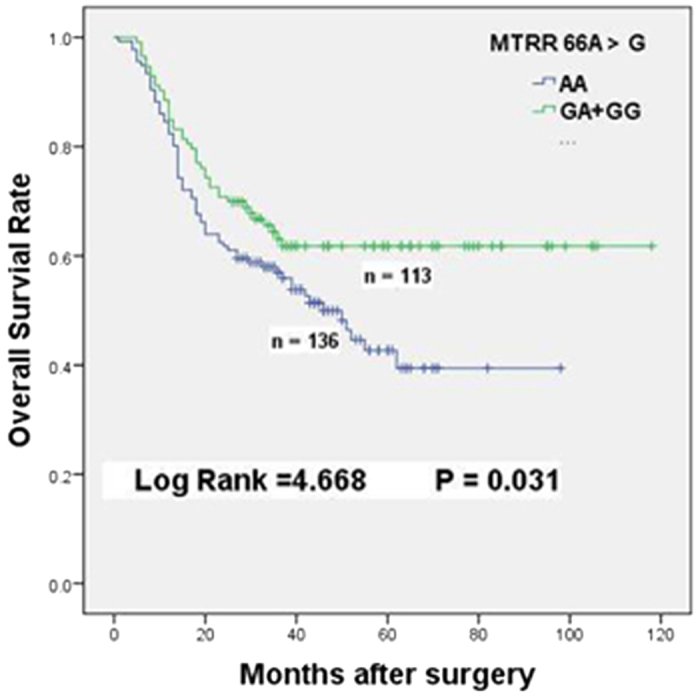
Overall survival of *MTRR66A*  > G dominant genotypes in GC patients receiving 5-FU-based chemotherapy.

Cox regression analyses were used to assess relationships of polymorphisms of those genes in the OCM pathway with overall survival in different genetic models among the two cohorts (Table 2). For the patients with adjuvant regimens, significant associations were found between MTRR 66A > G polymorphism and overall survival time in the dominant model. Chemotherapy patients with MTRR 66GA + GG genotypes (MST, 80 months) exhibited a 34.3% obviously lower risk of death (HR = 0.657, 95% CI = 0.446–0.967, p = 0.031, Table 2, Fig. 1) when compared with the patients with AA genotype (MST, 46 months), whereas no associations were founded among the cohort without chemotherapy.

For TS 5′-UTR 2R > 3R polymorphism, chemotherapy patients with 2R3R + 3R3R genotype (MST, 73 months) had a 50.2% notably lower risk of death (HR = 0.498, 95% CI = 0.259–0.960, p = 0.032, Table 2) when compared with those with 2R2R genotype (MST, 23 months). 5′-UTR 3R3R genotype alone also remarkably prolonged the survival time of chemotherapy patients (MST, 70 months; HR = 0.435, 95% CI = 0.221–0.857, p = 0.024).

For other polymorphisms including MTHFR 677C > T, 1298A > C MTR 2756A > G and TS3′-UTR 6 bp ins > del, no significant relationships were founded between polymorphisms in any genetic models and survival among patients accepted 5–FU treatment (Table 2). Furthermore, none of the six polymorphisms were correlated with overall survival for patients without 5-FU treatment.

### Stratified analyses of MTRR 66A > G polymorphism on reducing the death risk of GC patients accepted 5-FU-based chemotherapy

**Figure 2:**
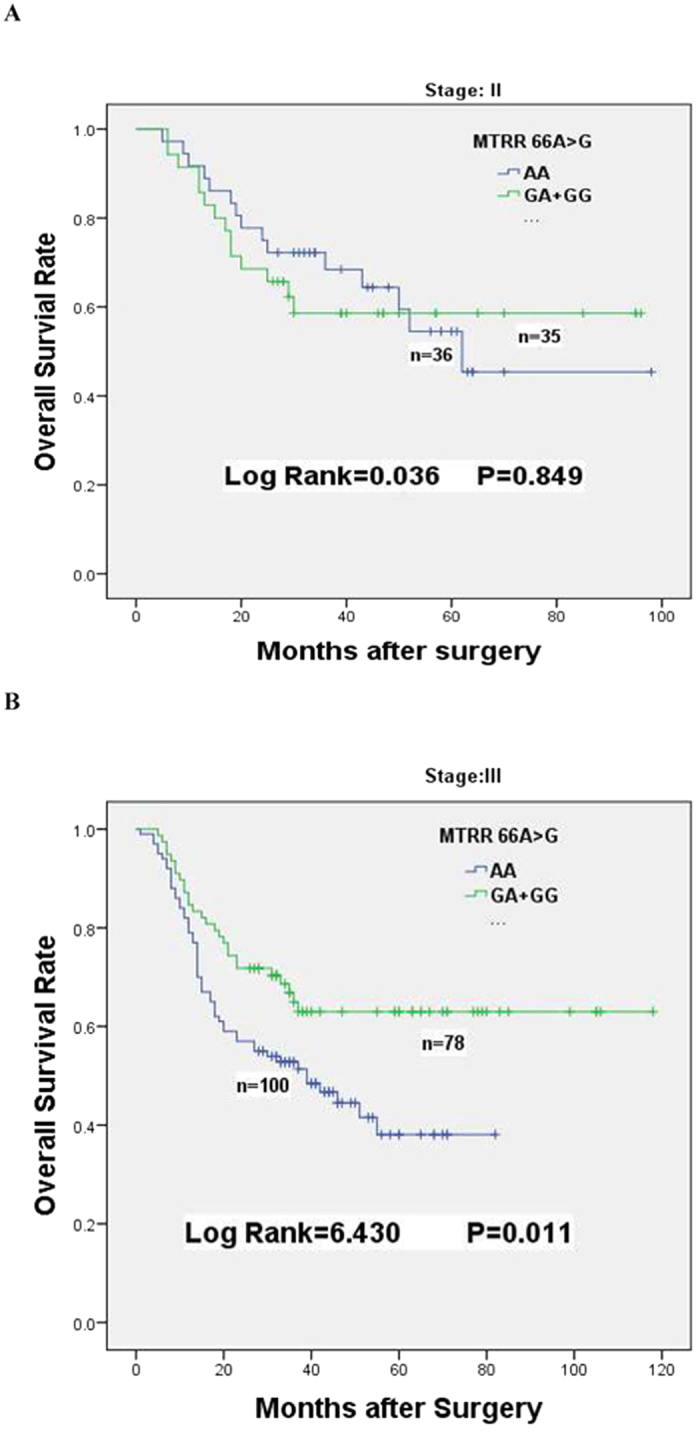
Kaplan-Meier survival curves of *MTRR66A* > G dominant genotypes for overall survival of GC patients received adjuvant chemotherapy. (**A**) Overall survival of *MTRR66A* > *G* dominant genotypes in stage II chemotherapy patients. (**B**) Overall survival of *MTRR66A* > *G* dominant genotypes in stage III chemotherapy patients.

To further explore the relationships between survival time and MTRR 66A > G polymorphism among GC patients with 5-FU-based chemotherapy, stratified analysis were conducted according to the histological types, sizes, sites, TNM stages, depth of invasion, lymph node metastasis, distant metastasis and chemotherapy (Table 3). Compared with the AA genotype, the MTRR 66 GA + GG genotypes were obviously associated with favorable survival of GC patients in diffuse type (MST, 83 months vs. 43 months; HR = 0.573; 95% CI: 0.356–0.923, p = 0.019), poorly differentiation (MST, 89 months vs. 50 months; HR = 0.460; 95% CI = 0.266–0.795, p = 0.004), T3/T4 level depth of invasion (MST, 82 months vs. 50 months; HR = 0.631, 95% CI = 0.414–0.961, p = 0.029), N1/N2/N3 lymph node metastasis (MST, 80 months vs. 39 months; HR = 0.562, 95% CI = 0.367–0.863, p = 0.007) and III level TNM stages (MST, 81 months vs. 39 months; HR = 0.557, 95% CI = 0.350–0.885, p = 0.01, Table 3, Fig. 2) .

**Figure 3:**
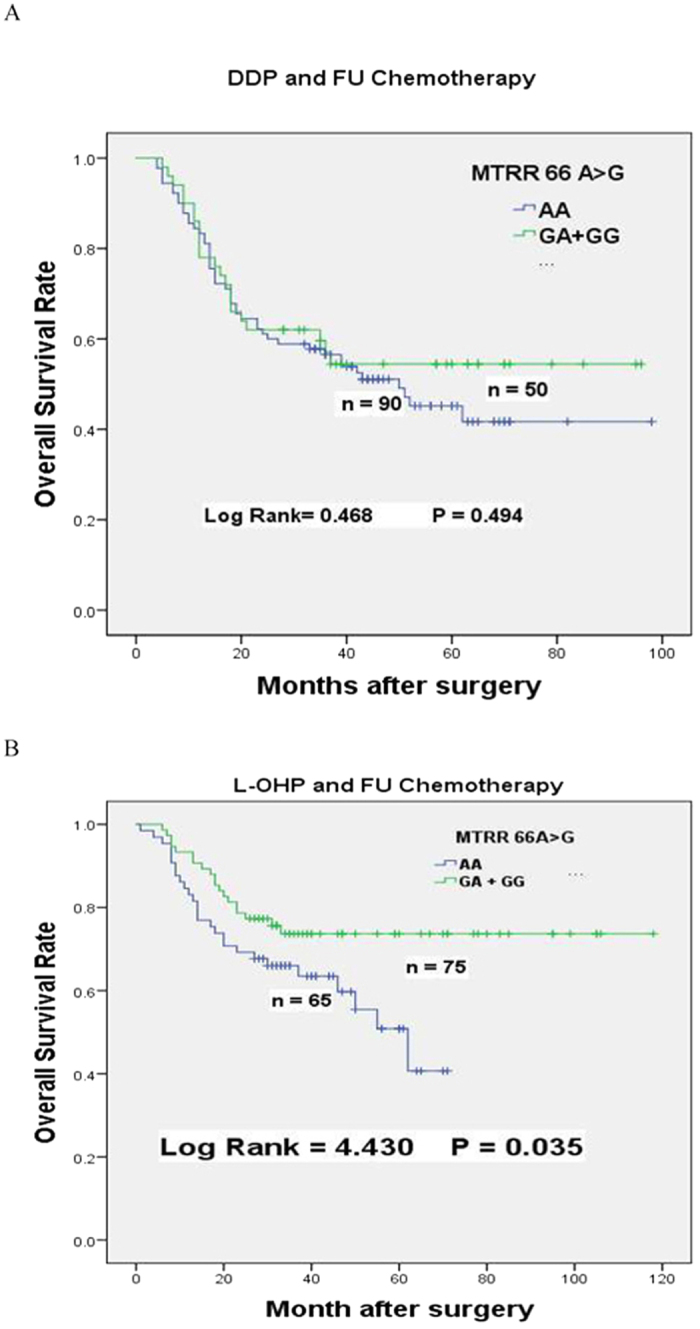
Kaplan-Meier survival curves of *MTRR66A* > G dominant genotypes for overall survival of GC patients received various chemotherapy regimen subgroups. (**A**) Overall survival of *MTRR66A* > *G* dominant genotypes patients treated with chemotherapy based on DDP and 5-FU. (**B**) Overall survival of *MTRR66A* > *G* dominant genotypes patients treated with chemotherapy based on L-OHP and 5-FU.

Furthermore, we stratified patients by chemotherapy regimens as combinations of DDP and 5-FU, or L-OHP and 5-FU subgroups. In dominant model, MTRR 66 GA + GG patients treated with L-OHP and 5-FU had favorable prognosis (MST, 92 months vs. 62 months; HR = 0.538, 95% CI = 0.299–0.970, p = 0.035, Table 4, Fig. 3) than those with AA genotype. Markedly, the death rate was reduced from 41.5% (27/65, patients with AA genotype) to 25.3% (19/75, patients with GA + GG genotype). Surprisingly, no statistically significant protection was observed in subgroup accepted chemotherapy based on DDP and 5-FU (HR = 0.839, 95% CI = 0.506–1.393, p = 0.494, Table 4, Fig. 3).

### The effects of combined polymorphisms on 5–FU-based chemotherapy

Considering the complicated interactions of the genes outlined above, we investigated the effect of polymorphism interactions on the prognosis of GC patients who accepted 5–FU treatment (Supplementary Tables S1–S3). In univariate analysis, MTRR 66 GA + GG genotypes remarkably reduced death risk, but the elongated survival time disappeared when GC patients simultaneously carried MTR 2756GA + GG genotypes (HR = 0.871, 95% CI = 0.443–1.713) or MTHFR 677TT +TC genotypes (HR = 0.761, 95% CI = 0.451–1.287, Table 5). In these cases, the MSTs were reduced from 80 months to 60 and 64 months, respectively. In addition, it seemed that patients simultaneous with TS 3′-UTR DD + DI and TS 5′-UTR 2R3R + 3R3R genotypes showed higher reduction of death risk (HR = 0.332, 95% CI = 0.134–0.822, log-rank p = 0.046, Table 5) than those with TS 5′-UTR 2R3R + 3R3R (HR = 0.498, 95% CI = 0.259–0.960) genotypes alone.

## Discussion

We systematically investigated the effects of SNPs in the OCM pathway and their interactions on the survival of stage II-III GC patients, who received 5-FU base adjuvant chemotherapy. Our results suggested that the polymorphisms of MTRR 66A > G and TS 5′-UTR 2R > 3R remarkably prolonged the survival of Chinese stage II-III GC patients treated with 5-FU base regimen. The MST of GC patients was also significantly influenced by interactions between MTRR 66A > G, MTR 2756A > G, TS 5′-UTR 2R > 3R and MTHFR 677C > T.

As an inhibitor of DNA synthesis, 5-FU displays potent anticancer activity by inhibiting TS activity through forming a ternary complex with 5, 10-MTHF^21^. The SNPs of MTRR 66 A > G and MTR 2756 A > G has been shown to decrease MTR enzyme activity. MTRR is used to maintain MTR in its active form, but the activity of MTR could not be preserved at a high level because the substitution of Ile22 to Met22 in MTRR caused by the 66A > G mutation decreased MTR activity^22^. The reduced MTR activity resulted in an accumulation of 5-MTHF. Subsequently, it increases the level of 5,10-MTHF that ultimately enhances the effect of 5-FU treatment^20^. Our result indicated that MTRR 66GA + GG genotypes significantly improved the survival of GC patients in the chemotherapy cohort (HR = 0.657, 95% CI = 0.446–0.967, Table 2, Fig. 1). This is consistent with other studies^23^,^24^. Further stratified analysis on pathological features showed that many factors, including histological type, tumor differentiation, depth of invasion, lymph node metastasis and TNM stages of patients, strongly influenced the survival of GC patients carrying MTRR 66GA + GG genotypes (Table 3). In the case of MTR 2756 A > G polymorphism, Sarbia *et al.* had indicated that patients with MTR 2756GA + GG genotypes had favorable outcomes when they underwent multimodal treatment including 5-FU-based radiochemotherapy and surgery^25^. However, we found that this polymorphism had no obvious association with prognosis of patients receiving 5-FU-based chemotherapy. In the combination analysis, although the favorable response was maintained when patients simultaneously carry MTRR 66GA + GG and MTR 2756AA genotypes; interestingly, MTR 2756GG + GA genotypes neutralized the protective effect of MTRR 66GA + GG (HR = 0.871, 95% CI = 0.443–1.713, Table 5). The similar neutralization was also existed when patients simultaneous with MTRR 66GG + GA and MTHFR 677TT + TC genotypes (Table 5). The neutralization maybe caused by the interactions of those key enzymes involved in the OCM pathway. Asp868 of *MTR* and Ile22 of *MTRR* may play vital role in the interactions between *MTRR* and *MTR*. Met22 conversion of MTRR may breakdown MTRR-MTR interaction, but Gly868 conversion of MTR may compensate and recover those interactions to a certain extent by changing the three dimensional spatial structure^26^. Similar interactions probably also exist between MTRR and MTHFR. Because our results were from a single cohort study with limited number of patients, the results would be validated in other cohorts with enlarged sample sizes.

The stratified analysis of different combined chemotherapy regimens showed that patients with MTRR 66GA + GG genotypes treated with L-OHP and 5-FU had remarkably favorable prognosis than those with AA genotypes (HR = 0.538, 95% CI = 0.299–0.970, p = 0.035, Table 4, Fig. 3). Whereas, no obviously improved survival was founded among patients receiving DDP and 5-FU chemotherapy (HR = 0.839, 95% CI = 0.506–1.393, p = 0.494, Table 4, Fig. 3). This is not surprising because L-OHP displays much more potent apoptotic activity than DDP^27^. Although L-OHP forms fewer intrastrand cross-links with adjacent guanines (GG) and occasionally adenines (AG) than DDP at equimolar concentrations, L-OHP-Pt-DNA adducts are more lethal than CDDP-Pt-DNA adducts^28^. The other reason might be differential DNA repair of L-OHP and DDP lesions. Intact mismatch repair is the main way for DDP induced cytotoxicity; whereas, L-OHP adducts are poorly recognized by mismatch repair proteins^29^,^30^,^31^.

A number of studies showed that the polymorphisms of TS affect the sensitivity to 5-FU, but the results were controversial. It was discovered that the 3R3R genotype was related with poor outcomes for colorectal cancer patients treated with 5-FU^32^,^33^. For GC patients receiving 5-FU regimens, Ott *et al.* also found that 3R3R was a risk factor for the survival^34^. Whereas, some other studies reported that no significant differences were observed for the survival of patients with different genotypes^35^,^36^. Jakobsen *et al.* reported that patients with 3R3R genotype responded better to 5-FU and had longer survival time^11^. Obviously, the effects of TS 5′-UTR polymorphism on 5-FU treatment are very complicated. The explanation for those controversies may involve in cancer type, genetic background and gene-gene interactions. Our finding was similar to Jakobsen’s result (HR = 0.498, 95% CI = 0.259–0.960, p = 0.032, Table 2). The 3R allele could enhance TS expression when compared with the 2R allele, and the higher TS expression level caused by 2R3R and 3R3R genotypes would increase the formation of the ternary complex, leading to the improvement of sensitivity to 5-FU. The inhibition of TS may give rise to insufficient dTMP supply in DNA synthesis, dUMP misincorporation into DNA and double-strand breaks followed by cell apoptosis^37^,^38^. Controversial data on TS 3′-UTR 6 bp ins > del polymorphism were also reported. In colorectal cancer patients receiving chemotherapy, some studies showed a better outcome of 6bp ins > del polymorphism on the survival^10^,^39^, while another report exhibited a higher response rate for patients with TS 6bp ins/ins genotype than those with TS 6bp del/del genotype^40^. Several other studies, similar to our findings, demonstrated that no obvious different survival times were identified among the patients with different genotypes^41^,^42^. Interestingly, although no obvious relativity was found between TS 3′-UTR 6bp ins > del polymorphism with patients′ survival, the protective effect of TS 5′-UTR 2R > 3R polymorphism was enhanced when chemotherapy patients simultaneous with TS 3′-UTR DI + DD and TS 5′-UTR 2R3R + 3R3R genotypes (HR = 0.332, 95% CI = 0.134–0.822, log-rank p = 0.046, Table 5). The interaction between TS 5′-UTR and 3′-UTR may be the explanation for this synergistic effect.

The 677 C > T polymorphism induces an Ala-to-Val switch at codon 222 in MTHFR, resulting in thermolabile variant of the enzyme with reduced activity^43^. The substitution of Glu-to-Ala at codon 429 in MTHFR caused by 1298 A > C mutation also reduced enzyme activity, but at a lower degree compared to 677 C > T^43^,^44^. Although decreased MTHFR activity improve the chemotherapy sensitivity of 5-FU by increasing the level of 5,10-MTHF, the reported data were also inconsistent. For the chemotherapy cohort, some groups reported that MTHFR 1298 A > G and 2756 C > T polymorphism responded negatively to 5-FU for colorectal and gastric cancers patients^45^,^46^,^47^. Whereas, Omar *et al.* showed that the polymorphism of the MTHFR 677C > T was associated with longer TTP and OS for chemotherapy patients^48^. Some reported that no remarkable relationship was found between MTHFR 1298 A > G and 2756 C > T polymorphisms and patient’s survival^12^,^49^,^50^, which was consistent with our results. The inconsistent results may be caused by diverse schedules, the varied doses of 5-FU used, various cancer stages or types, and various ethnic populations among those studies.

In conclusion, our study systematically investigated the effects of polymorphisms and their interactions of genes in OCM pathway on clinical prognosis of stage II-III GC patients treated with 5-FU-based regimens. Our findings showed that the polymorphisms of MTRR 66A > G, TS 5′-UTR 2R > 3R may serve as potential prognostic markers for GC patients receiving 5-FU-based regimens. Results from this study indicated that gene-gene interactions may enhance or neutralize a drug’s anticancer effects, emphasizing that further investigations should be conducted in different cohorts with larger number of patients.

### Patients and methods

#### Patient samples

From January 1999 to December 2006, 650 stage II-III patients histopathologically diagnosed with GC were recruited in our study. They underwent surgery at the Yixing People’s Hospital (Yixing, Jiangsu Province, China). None of them had received chemotherapy or radiotherapy before operation. 251 patients had 5-FU-based adjuvant chemotherapy, and the other 399 patients had not due to various reasons. The chemotherapy regimens were 5-FU in combination with DDP or L-OHP. Chemotherapy was not conducted until the patients met the following characteristics: platelet counts ≥100 × 10^9^/L, neutrophil counts ≥1.5 × 10^9^/L, hemoglobin level ≥8 g/dl and no sign of organ toxicity. The general clinical characteristics of patients were summarized in Table 1. The median follow-up time is 68.5 months and the maximum follow-up time is 119.0 months. According to Lauren’s Standard, the histopathology of GC was classified into intestinal or diffuse types^51^. The TNM stages were evaluated according to the TNM classification of the American Joint Committee on Cancer (AJCC cancer staging manual, seventh edition). This study was approved by the Institutional Review Board of Nanjing Medical University (Nanjing, China). And the methods were carried out in accordance with the approved guidelines. All patients enrolled in this study signed an informed consent on using clinical specimen in medical research.

#### Genotyping

Genomic DNA was extracted from tumor specimens as described previously^23^. Polymorphisms of the above genes were determined by SNaPshot technology with either ABI florescence-based assay allelic discrimination assay (Applied Biosystems, Forster City, CA) or automated sequencing^52^. The primers used for sequencing in this study are listed in Supplementary Table S4. Genotypes were determined by Genemapper 4.0 software (Applied Biosystems) and the polymorphisms were analyzed in ABI3130 genetic analyzer. About 10% samples were randomly selected to validate genotypes by two people independently. The results were 100% concordant.

#### Statistical analysis

The overall survival time was calculated from the date of surgery to the last follow up time or death (March, 2009) and mean survival time was chosen if the median survival time (MST) could not be calculated. The relationships of each genotype and combinations of genotypes with clinicopathologic characteristics were elucidated using Student t-test and the Pearson chi-square test according to the types of variables. Log-rank tests and Kaplan–Meier plots were performed by SPSS version 20.0 with two-sided tests and p < 0.05 was considered statistically significant (SPSS Inc, Chicago, IL, USA). Univariate or multivariate Cox regression analysis was adopted to estimate adjusted hazard ratios (HRs) and 95% confidence intervals (CIs).

## Additional Information

**How to cite this article**: Zhao, T. *et al.* The effects of genomic polymorphisms in one-carbon metabolism pathways on survival of gastric cancer patients received fluorouracil-based adjuvant therapy. *Sci. Rep.* **6**, 28019; doi: 10.1038/srep28019 (2016).

## Supplementary Material

Supplementary Information

## Figures and Tables

**Table 1:** Characteristics of the two cohorts of the gastric cancer patients.

Variable	Chemotherapy (251)				No chemotherapy (399)			
	Patients/deaths	MST (mouths)	P	HR (95% CI)^a^	Patients/deaths	MST (mouths)	p	HR (95% CI)^a^
age(years)								
<=60	144/60	67^b^	0.264	1	161/90	43	0.888	1
>60	107/50	50		1.236 (0.849–1.801)	138/128	50		1.020 (0.777–1.337)
Sex								
Male	205/91	70^b^	0.694	1	301/159	50	0.304	1
Female	46/19	55		0.906 (0.553–1.486)	98/59	36		1.168 (0.866–1.576)
Tumor size								
<=5	141/59	73^b^	0.304	1	211/111	53	0.223	1
>5	110/51	55		1.215 (0.835–1.768)	188/107	47		1.178 (0.903–1.537)
Tumor site								
No–cardia	157/69	64^b^	0.782	1	253/142	38	0.386	1
Cardia	94/41	55		0.947 (0.644–1.394)	146/76	59		0.885 (0.669–1.170)
Histological type								
Intestinal	73/35	62	0.417	1	143/75	42	0.306	1
Diffuse	178/75	72^b^		1.179(0.789–1.762)	256/143	54		1.158(0.872–1.537)
Tumor differentiation								
Well	68/33	51	0.148	1	120/50	74	0.038	1
Poorly	151/59	75^b^		0.742 (0.484–1.137)	231/137	38		1.436 (1.037–1.989)
Mucinous or signet-ring cell	32/18	45^b^		1.171(0.659–2.079)	47/30	35		1.675(1.064–2.636)
Depth of invasion								
T1/T2	24/13	35	0.390	1	43/19	68^b^	0.168	1
T3/T4	227/97	72^b^		0.778 (0.436–1.388)	351/195	48		1.388 (0.866–2.226)
Lymph node metastasis								
NO	52/18	69^b^	0.101	1	88/38	67	0.021	1
N1/N2/N3	199/92	62		1.518 (0.915–2.517)	308/178	38		1.500 (1.056–2.129)
TNM stage								
II	72/29	64^b^	0.325	1	126/51	71^b^	<0.001	1
III	179/81	69^b^		1.235 (0.808–1.888)	273/167	36		1.809 (1.322–2.477)

^a^Adjusted for age and sex.

^b^Mean survival time was provided when MST could not be calculated.

^c^Information was not available for two patients.

HR, hazard ratio; CI, confidence interval; MST, median survival time; P, Log-rank p.

**Table 2:** Genotypes of *MTRR, MTHFR, MTR* and *TS* polymorphism with gastric cancer patients’ survival in both cohorts.

Genetic model	Genotypes	Chemotherapy			No chemotherapy		
		MST (mouths)	P	HR (95% CI)^a^	MST (mouths)	p	HR (95% CI)^a^
*MTRR rs1801394 66 A > G*							
Codominant model	AA	46	0.089	1	38	0.419	1
	GA	72^b^		0.675 (0.452–1.007)	62		0.880 (0.659–1.176)
	GG	85^b^		0.526 (0.192–1.443)	62		0.683 (0.347–1.344)
Dominant model	GA/GG	80^b^	0.031	0.657 (0.446–0.967)	62		0.853 (0.645–1.129)
*TYMS 5-UTR 2R > 3R*							
Codominant model	2R2R	23	0.024	1	60^b^	0.600	1
	2R3R	51		0.629 (0.312–1.268)	50		1.433 (0.658–3.124)
	3R3R	70^b^		0.435 (0.221–0.857)	50		1.474 (0.687–3.164)
Dominant model	2R3R/3R3R	73^b^	0.032	0.498 (0.259–0.960)	50	0.320	1.459 (0.685–3.106)
Recessive model	2R2R/2R3R	39	0.022	1	56	0.643	1
	3R3R	70^b^		0.637 (0.430–0.944)	50		1.071 (0.800–1.433)
*MTHFR rs1801131 1298 A > C*							
Codominant model	AA	62	0.55	1	48	0.739	1
	CA	72^b^		1.011 (0.656–1.559)	48		1.012 (0.747–1.371)
	CC	69^b^		0.471(0.116–1.918)	68^b^		0.585 (0.145–2.362)
Dominant model	CA/CC	75^b^	0.771	0.940 (0.616–1.433)	48	0.934	0.987 (0.732–1.333)
*MTHFR rs1801133 677C > T*							
Codominant model	CC	72^b^	0.697	1	38	0.610	1
	TC	59^b^		1.169 (0.771–1.774)	43		0.973 (0.694–1.266)
	TT	55		0.983 (0.555–1.741)	63		0.814 (0.541–1.226)
Dominant model	TC/TT	60^b^	0.587	1.118 (0.753–1.659)	48		0.904 (0.679–1.203)
*MTR rs1805087 2756 A > G*							
Codominant model	AA	62	0.613	1	53	0.059	1
	GA	73		0.943 (0.559–1.591)	42		1.3699 (0.957–1.958)
	GG	0		0			0
Dominant model	GA/GG	64^b^	0.681	0.897 (0.532–1.513)	26	0.205	1.257 (0.879–1.797)
*TYMS 3-UTR 6bp ins > del*							
Codominant model	I/I	46	0.171	1	55^b^	0.811	1
	D/I	50		1.028 (0.539–1.961)	47		1.207 (0.677–2.151)
	D/D	71^b^		0.703 (0.359–1.376)	50		1.192 (0.668–2.128)
Dominant model	DI/DD	72^b^	0.654	0.868 (0.456–1.620)	48	0.521	1.200 (0.684–2.104)

^a^Adjusted for age and sex.

^b^Mean survival time was provided when MST could not be calculated.

2R, 2-repeat; 3R, 3-repeat; I, 6-bp insertion; D; 6-bp deletion; P, Log-rank p.

**Table 3:** Stratified analysis of the *MTRR 66A* > *G* polymorphism with gastric cancer overall survival in patients received adjuvant chemotherapy.

Variables	Genotypes (Dominant model)		MST (AA/GA + GG)	HR (95% CI)^a^	*P*_*Heterogeneity*_
	AA	GA/GG			
Total	136/69	113/41	46/80^b^	0.657 (0.446–0.967)	0.031
Age					
≤60	73/35	69/25	52/73^b^	0.697 (0.416–1.165)	0.163
>60	63/34	44/16	42/79^b^	0.622 (0.343–1.127)	0.109
Tumor size					
≤5 cm	77/38	64/21	46/84^b^	0.607 (0.356–1.036)	0.062
>5 cm	59/31	49/20	51/67^b^	0.725 (0.413–1.273)	0.255
Tumor site					
Non-cardia	88/44	68/25	43/72^b^	0.645 (0.394–1.054)	0.076
Cardia	48/25	45/16	51/80^b^	0.700 (0.373–1.311)	0.259
Histological type					
Intestinal type	40/20	32/15	51/58^b^	0.891 (0.456–1.743)	0.734
Diffuse type	96/49	81/26	43/83^b^	0.573 (0.356–0.923)	0.019
Tumor differentiation					
Well to moderately	37/19	30/14	46/57^b^	0.864 (0.433–1.724)	0.676
poor	80/40	71/19	50/89^b^	0.460 (0.266–0.795)	0.004
mucinous /signet-ring cell	19/10	12/8	43/20	1.514 (0.592–3.871)	0.382
Depth of invasion					
T1/T2	8/5	16/8	24/56^b^	0.681 (0.222–2.088)	0.495
T3/T4	128/64	97/33	50/82^b^	0.631 (0.414–0.961)	0.029
Lymph node metastasis					
N0	29/10	22/8	70b/65^b^	1.276 (0.502–3.244)	0.606
N1/N2/N3	107/59	91/33	39/80^b^	0.562 (0.367–0.863)	0.007
Distant metastasis					
M0	136/69	113/41	46/80^b^	0.657 (0.446–0.967)	0.031
TNM stage					
II	36/15	35/14	62/63^b^	1.073 (0.517–2.226)	0.849
III	100/54	78/27	39/81^b^	0.557 (0.350–0.885)	0.011

^a^Adjusted for age and sex.

^b^Heterogeneity test for differences between groups.

^c^Information was not available for two patients.

**Table 4:** Association between the *MTRR 66A* > *G* dominant model and survival of gastric cancer patients among various chemotherapy regimen subgroups.

*MTRR66A* > *G*					
**Chemotherapy Based on DDP and 5-FU**					
**Genotype**	**Patients, n = 140**	**Deaths, n = 69**	**MST (months)**	**log-rank p**	**HR (95% CI)^a^**
AA	90	47	50	0.494	1
GA/GG	50	22	60^b^		0.839 (0.506–1.393)
**Chemotherapy Based on L-OHP and 5-FU**					
**Genotype**	**patients, n = 140**	**Deaths, n = 46**	**MST (months)**	**log-rank p**	**HR (95% CI)^a^**
AA	65	27	62	0.035	1
GA/GG	75	19	92^b^		0.538 (0.299–0.970)

^a^Adjusted for age and sex.

^b^Mean survival time was provided when MST could not be calculated.

**Table 5:** The effects of gene-gene interactions on the survival of gastric cancer patients received adjuvant chemotherapy.

Combined genotypes	Patients	Deaths	MST (months)	P	HR (95%CI)^a^
*MTRR 66A > G and MTR 2756A > G*					
*MTRR 66AA* + *MTR 2756AA*	105	54	43	0.149	1
*MTRR 66AA* + *MTR 2756GG* + *GA*	19	7	65^b^		0.631 (0.287–1.388)
*MTRR 66GG* + *GA* + *MTR 2756AA*	82	28	81^b^		0.607 (0.384–0.959)
*MTRR 66GG* + *GA* + *MTR 2756GG* + *GA*	21	10	60^b^		0.871 (0.443–1.713)
*MTRR 66A > G and MTHFR 677C > T*					
*MTRR 66AA* + *MTHFR 677CC*	57	27	50	0.133	1
*MTRR 66AA* + *MTHFR 677TT* + *TC*	77	41	43		1.146 (0.705–1.864)
*MTRR 66GG* + *GA* + *MTHFR 677CC*	34	11	85^b^		0.588 (0.291–1.187)
*MTRR 66GG* + *GA* + *MTHFR 677TT* + *TC*	77	29	64^b^		0.761 (0.451–1.287)
*TS3-UTR I > D and TS5-UTR 2R > 3R*					
*TS*3-UTR II + *TS*5-UTR 2R2R	5	5	18	0.046	1
*TS*3-UTR II+ *TS*5-UTR 2R3R + 3R3R	16	5	72^b^		0.223 (0.064–0.776)
*TS*3-UTR DD + DI + *TS*5-UTR 2R2R	9	5	39		0.481 (0.139–1.666)
*TS*3-UTRDD + DI+ *TS*5-UTR 2R3R + 3R3R	202	85	73^b^		0.332 (0.134–0.822)

^a^Adjusted for age and sex.

^b^Mean survival time was provided when MST could not be calculated.

## References

[b1] Siegel R., Naishadham D. & Jemal A. Cancer statistics, 2013. CA Cancer J Clin 63, 11–30, doi: 10.3322/caac.21166 (2013). 23335087

[b2] Wang Y. C., Wei L. J., Liu J. T., Li S. X. & Wang Q. S. Comparison of Cancer Incidence between China and the USA. Cancer Biol Med 9, 128–132, doi: 10.3969/j.issn.2095-3941.2012.02.009 (2012). 23691468 PMC3643656

[b3] Wagner A. D. *et al.* Chemotherapy in advanced gastric cancer: a systematic review and meta-analysis based on aggregate data. J Clin Oncol 24, 2903–2909, doi: 10.1200/JCO.2005.05.0245 (2006). 16782930

[b4] Group G. *et al.* Benefit of adjuvant chemotherapy for resectable gastric cancer: a meta-analysis. JAMA 303, 1729–1737, doi: 10.1001/jama.2010.534 (2010). 20442389

[b5] Kang B. W., Kim J. G., Kwon O. K., Chung H. Y. & Yu W. Non-platinum-based chemotherapy for treatment of advanced gastric cancer: 5-fluorouracil, taxanes, and irinotecan. World J Gastroenterol 20, 5396–5402, doi: 10.3748/wjg.v20.i18.5396 (2014). 24833869 PMC4017054

[b6] Modulation of fluorouracil by leucovorin in patients with advanced colorectal cancer: evidence in terms of response rate. Advanced Colorectal Cancer Meta-Analysis Project. J Clin Oncol 10, 896–903 (1992). 1534121 10.1200/JCO.1992.10.6.896

[b7] Naushad S. M., Pavani A., Digumarti R. R., Gottumukkala S. R. & Kutala V. K. Epistatic interactions between loci of one-carbon metabolism modulate susceptibility to breast cancer. Mol Biol Rep 38, 4893–4901, doi: 10.1007/s11033-010-0631-z (2011). 21161404

[b8] Longley D. B., Harkin D. P. & Johnston P. G. 5-fluorouracil: mechanisms of action and clinical strategies. Nat Rev Cancer 3, 330–338, doi: 10.1038/nrc1074 (2003). 12724731

[b9] Kuhn J. G. Fluorouracil and the new oral fluorinated pyrimidines. Ann Pharmacother 35, 217–227 (2001). 11215843 10.1345/aph.10096

[b10] Huang Z. H., Hua D. & Li L. H. The polymorphisms of TS and MTHFR predict survival of gastric cancer patients treated with fluorouracil-based adjuvant chemotherapy in Chinese population. Cancer Chemother Pharmacol 63, 911–918, doi: 10.1007/s00280-008-0815-6 (2009). 18704422

[b11] Jakobsen A., Nielsen J. N., Gyldenkerne N. & Lindeberg J. Thymidylate synthase and methylenetetrahydrofolate reductase gene polymorphism in normal tissue as predictors of fluorouracil sensitivity. J Clin Oncol 23, 1365–1369, doi: 10.1200/JCO.2005.06.219 (2005). 15735113

[b12] Afzal S. *et al.* MTHFR polymorphisms and 5-FU-based adjuvant chemotherapy in colorectal cancer. Ann Oncol 20, 1660–1666, doi: 10.1093/annonc/mdp046 (2009). 19465420

[b13] Vanhoefer U. *et al.* Final results of a randomized phase III trial of sequential high-dose methotrexate, fluorouracil, and doxorubicin versus etoposide, leucovorin, and fluorouracil versus infusional fluorouracil and cisplatin in advanced gastric cancer: A trial of the European Organization for Research and Treatment of Cancer Gastrointestinal Tract Cancer Cooperative Group. J Clin Oncol 18, 2648–2657 (2000). 10894863 10.1200/JCO.2000.18.14.2648

[b14] Kawakami K. *et al.* Different lengths of a polymorphic repeat sequence in the thymidylate synthase gene affect translational efficiency but not its gene expression. Clin Cancer Res 7, 4096–4101 (2001). 11751507

[b15] Mandola M. V. *et al.* A 6 bp polymorphism in the thymidylate synthase gene causes message instability and is associated with decreased intratumoral TS mRNA levels. Pharmacogenetics 14, 319–327 (2004). 15115918 10.1097/00008571-200405000-00007

[b16] Marsh S., McKay J. A., Cassidy J. & McLeod H. L. Polymorphism in the thymidylate synthase promoter enhancer region in colorectal cancer. Int J Oncol 19, 383–386 (2001). 11445856 10.3892/ijo.19.2.383

[b17] Etienne M. C. *et al.* Methylenetetrahydrofolate reductase gene polymorphisms and response to fluorouracil-based treatment in advanced colorectal cancer patients. Pharmacogenetics 14, 785–792 (2004). 15608557 10.1097/00008571-200412000-00001

[b18] Lu J. W. *et al.* [Relationship of methylenetetrahydrofolate reductase C677T polymorphism and chemosensitivity to 5-fluorouracil in gastric carcinoma]. Ai Zheng 23, 958–962 (2004). 15301724

[b19] Lissowska J. *et al.* Genetic polymorphisms in the one-carbon metabolism pathway and breast cancer risk: a population-based case-control study and meta-analyses. Int J Cancer 120, 2696–2703, doi: 10.1002/ijc.22604 (2007). 17311260

[b20] Laraqui A. *et al.* Influence of methionine synthase (A2756G) and methionine synthase reductase (A66G) polymorphisms on plasma homocysteine levels and relation to risk of coronary artery disease. Acta Cardiol 61, 51–61 (2006). 16485733 10.2143/AC.61.1.2005140

[b21] Blank S. *et al.* A retrospective comparative exploratory study on two methylentetrahydrofolate reductase (MTHFR) polymorphisms in esophagogastric cancer: the A1298C MTHFR polymorphism is an independent prognostic factor only in neoadjuvantly treated gastric cancer patients. BMC Cancer 14, 58, doi: 10.1186/1471-2407-14-58 (2014). 24490800 PMC3922603

[b22] Olteanu H., Munson T. & Banerjee R. Differences in the efficiency of reductive activation of methionine synthase and exogenous electron acceptors between the common polymorphic variants of human methionine synthase reductase. Biochemistry 41, 13378–13385 (2002). 12416982 10.1021/bi020536s

[b23] Wang M. *et al.* Genetic variant in PSCA predicts survival of diffuse-type gastric cancer in a Chinese population. Int J Cancer 129, 1207–1213, doi: 10.1002/ijc.25740 (2011). 21064099

[b24] Luo D. *et al.* Genetic variation in PLCE1 is associated with gastric cancer survival in a Chinese population. J Gastroenterol 46, 1260–1266, doi: 10.1007/s00535-011-0445-3 (2011). 21837401

[b25] Sarbia M., Stahl M., von Weyhern C., Weirich G. & Puhringer-Oppermann F. The prognostic significance of genetic polymorphisms (Methylenetetrahydrofolate Reductase C677T, Methionine Synthase A2756G, Thymidilate Synthase tandem repeat polymorphism) in multimodally treated oesophageal squamous cell carcinoma. Br J Cancer 94, 203–207, doi: 10.1038/sj.bjc.6602900 (2006). 16333305 PMC2361119

[b26] Zhao T. *et al.* Polymorphism in one-carbon metabolism pathway affects survival of gastric cancer patients: Large and comprehensive study. Oncotarget 6, 9564–9576, doi: 10.18632/oncotarget.3259 (2015). 25840420 PMC4496239

[b27] Faivre S., Chan D., Salinas R., Woynarowska B. & Woynarowski J. M. DNA strand breaks and apoptosis induced by oxaliplatin in cancer cells. Biochem Pharmacol 66, 225–237 (2003). 12826265 10.1016/s0006-2952(03)00260-0

[b28] Virag P. *et al.* Superior cytotoxicity and DNA cross-link induction by oxaliplatin versus cisplatin at lower cellular uptake in colorectal cancer cell lines. Anticancer Drugs 23, 1032–1038, doi: 10.1097/CAD.0b013e328355076f (2012). 22614106

[b29] Vaisman A. *et al.* The role of hMLH1, hMSH3, and hMSH6 defects in cisplatin and oxaliplatin resistance: correlation with replicative bypass of platinum-DNA adducts. Cancer Res 58, 3579–3585 (1998). 9721864

[b30] Ferry K. V. *et al.* Decreased cisplatin damage-dependent DNA synthesis in cellular extracts of mismatch repair deficient cells. Biochem Pharmacol 57, 861–867 (1999). 10086318 10.1016/s0006-2952(98)00366-9

[b31] Fink D. *et al.* The role of DNA mismatch repair in platinum drug resistance. Cancer Res 56, 4881–4886 (1996). 8895738

[b32] Iacopetta B., Grieu F., Joseph D. & Elsaleh H. A polymorphism in the enhancer region of the thymidylate synthase promoter influences the survival of colorectal cancer patients treated with 5-fluorouracil. Br J Cancer 85, 827–830, doi: 10.1054/bjoc.2001.2007 (2001). 11556832 PMC2375084

[b33] Martinez-Balibrea E. *et al.* Combined analysis of genetic polymorphisms in thymidylate synthase, uridine diphosphate glucoronosyltransferase and X-ray cross complementing factor 1 genes as a prognostic factor in advanced colorectal cancer patients treated with 5-fluorouracil plus oxaliplatin or irinotecan. Oncol Rep 17, 637–645 (2007). 17273745

[b34] Ott K. *et al.* The thymidylate synthase tandem repeat promoter polymorphism: A predictor for tumor-related survival in neoadjuvant treated locally advanced gastric cancer. Int J Cancer 119, 2885–2894, doi: 10.1002/ijc.22235 (2006). 16929515

[b35] Hua D., Huang Z. H., Mao Y. & Deng J. Z. Thymidylate synthase and thymidine phosphorylase gene expression as predictive parameters for the efficacy of 5-fluorouracil-based adjuvant chemotherapy for gastric cancer. World J Gastroenterol 13, 5030–5034 (2007). 17854149 10.3748/wjg.v13.i37.5030 PMC4434630

[b36] Ruzzo A. *et al.* Pharmacogenetic profiling in patients with advanced colorectal cancer treated with first-line FOLFOX-4 chemotherapy. J Clin Oncol 25, 1247–1254, doi: 10.1200/JCO.2006.08.1844 (2007). 17401013

[b37] Canman C. E., Tang H. Y., Normolle D. P., Lawrence T. S. & Maybaum J. Variations in patterns of DNA damage induced in human colorectal tumor cells by 5-fluorodeoxyuridine: implications for mechanisms of resistance and cytotoxicity. Proc Natl Acad Sci USA 89, 10474–10478 (1992). 1438236 10.1073/pnas.89.21.10474 PMC50361

[b38] Yoshioka A. *et al.* Deoxyribonucleoside triphosphate imbalance. 5-Fluorodeoxyuridine-induced DNA double strand breaks in mouse FM3A cells and the mechanism of cell death. J Biol Chem 262, 8235–8241 (1987). 2954951

[b39] Lu J. W. *et al.* Polymorphism in the 3′-untranslated region of the thymidylate synthase gene and sensitivity of stomach cancer to fluoropyrimidine-based chemotherapy. J Hum Genet 51, 155–160, doi: 10.1007/s10038-005-0339-4 (2006). 16424979

[b40] Shitara K. *et al.* Folate intake along with genetic polymorphisms in methylenetetrahydrofolate reductase and thymidylate synthase in patients with advanced gastric cancer. Cancer Epidemiol Biomarkers Prev 19, 1311–1319, doi: 10.1158/1055-9965.EPI-09-1257 (2010). 20447923

[b41] Jang M. J. *et al.* Polymorphisms of folate metabolism-related genes and survival of patients with colorectal cancer in the Korean population. Gene 533, 558–564, doi: 10.1016/j.gene.2013.09.056 (2014). 24100087

[b42] Kumamoto K. *et al.* Polymorphisms of , and -3′UTR are associated with the clinical outcome of mFOLFOX6 in colorectal cancer patients. Oncol Lett 6, 648–654, doi: 10.3892/ol.2013.1467 (2013). 24137384 PMC3789107

[b43] Bottiger A. K., Hurtig-Wennlof A., Sjostrom M., Yngve A. & Nilsson T. K. Association of total plasma homocysteine with methylenetetrahydrofolate reductase genotypes 677C > T, 1298A > C, and 1793G > A and the corresponding haplotypes in Swedish children and adolescents. Int J Mol Med 19, 659–665 (2007). 17334642

[b44] Weisberg I., Tran P., Christensen B., Sibani S. & Rozen R. A second genetic polymorphism in methylenetetrahydrofolate reductase (MTHFR) associated with decreased enzyme activity. Mol Genet Metab 64, 169–172, doi: 10.1006/mgme.1998.2714 (1998). 9719624

[b45] Fernandez-Peralta A. M. *et al.* Association of polymorphisms MTHFR C677T and A1298C with risk of colorectal cancer, genetic and epigenetic characteristic of tumors, and response to chemotherapy. Int J Colorectal Dis 25, 141–151, doi: 10.1007/s00384-009-0779-y (2010). 19669769

[b46] Gao C. M. *et al.* [Polymorphism of methylenetetrahydrofolate reductase and sensitivity of stomach cancer to fluoropyrimidine-based chemotherapy]. Zhonghua Liu Xing Bing Xue Za Zhi 25, 1054–1058 (2004). 15769364

[b47] Cohen V. *et al.* Methylenetetrahydrofolate reductase polymorphism in advanced colorectal cancer: a novel genomic predictor of clinical response to fluoropyrimidine-based chemotherapy. Clin Cancer Res 9, 1611–1615 (2003). 12738713

[b48] Castillo-Fernandez O. *et al.* Methylenetetrahydrofolate reductase polymorphism (677 C > T) predicts long time to progression in metastatic colon cancer treated with 5-fluorouracil and folinic acid. Arch Med Res 41, 430–435, doi: 10.1016/j.arcmed.2010.08.011 (2010). 21044746

[b49] Zhu L. *et al.* Association between MTHFR polymorphisms and overall survival of colorectal cancer patients in Northeast China. Med Oncol 30, 467, doi: 10.1007/s12032-013-0467-1 (2013). 23392576

[b50] Gusella M. *et al.* Predictors of survival and toxicity in patients on adjuvant therapy with 5-fluorouracil for colorectal cancer. Br J Cancer 100, 1549–1557, doi: 10.1038/sj.bjc.6605052 (2009). 19384296 PMC2696766

[b51] Lauren P. The Two Histological Main Types of Gastric Carcinoma: Diffuse and So-Called Intestinal-Type Carcinoma. An Attempt at a Histo-Clinical Classification. Acta Pathol Microbiol Scand 64, 31–49 (1965). 14320675 10.1111/apm.1965.64.1.31

[b52] Xiao H. W. *et al.* Relationship between TNFA, TNFB and TNFRII gene polymorphisms and outcome after unrelated hematopoietic cell transplantation in a Chinese population. Bone Marrow Transplant 46, 400–407, doi: 10.1038/bmt.2010.135 (2011). 20548340

