# Supplementary material for: The effects of genomic polymorphisms in one-carbon metabolism pathways on survival of gastric cancer patients received fluorouracil-based adjuvant therapy
Source: Sci Rep. 2016 Jul 26;6:28019. doi: 10.1038/srep28019 (PMC4960563; doi:10.1038/srep28019)
Supplement: Supplementary Information [file srep28019-s1.doc]

**The effects of genomic polymorphisms in one-carbon metabolism pathways on survival of gastric cancer patients received fluorouracil-based adjuvant therapy**

Tingting Zhao^1,*^, Zhi Xu^1,*^, Dongying Gu^1,*^, Peng Wu^1*^, Xinying Huo^1^, Xiaowei Wei^1^, Yongfei Tang^2^, Weida Gong^3^, Ming-Liang He^4, 5^ & Jinfei Chen^1, 6^

^1^Department of Oncology, Nanjing First Hospital, Nanjing Medical University, Nanjing, 210006, China

^2^Department of Surgery, Yixing People’s Hospital, Yixing, 214200, China

^3^Department of Surgery, Yixing Cancer Hospital, Yixing, 214200, China
^4^Department of Biomedical Sciences, City University of Hong Kong, Hong Kong

^5^ CUHK Shenzhen Research Institute, Shenzhen, 518000, China

^6^Collaborative Innovation Center for Cancer Personalized Medicine, Nanjing Medical University, Nanjing, China.

*These authors contributed equally to this work.

**Correspondence to:**
Jinfei Chen, **e-mail:** jinfeichen@sohu.com
Ming-Liang He, **e-mail:** [mlhe7788@gmail.com](mailto:mlhe7788@gmail.com)

**Key words:** 5-fluorouracil (5-FU), adjuvant chemotherapy, gastric cancer (GC), single nucleotide polymorphism (SNP), one-carbon metabolism (OCM).

**Supplementary Table S1**. The effects of gene-gene interactions in homozygous model on the survival of gastric cancer patients receiving 5-FU–based chemotherapy

| **combined genotypes** | **patient** | **death** | **MST** | **P** | **HR (95%CI)^a^** |
| --- | --- | --- | --- | --- | --- |
| ***MTRR 66A>G and MTHFR 1298A>C*** | | | | | |
| *MTRR 66AA + MTHFR 1298AA* | 95 | 49 | 46 | 0.611 | 1 |
| *MTRR 66AA + MTHFR 1298CC* | 4 | 2 | 20 |  | 0.987(0.239-4.076) |
| *MTRR 66GG + MTHFR 1298AA* | 10 | 4 | 55b |  | 0.601(0.216-1.674) |
| *MTRR 66GG + MTHFR 1298CC* |  |  |  |  |  |
| ***MTRR 66A>G and MTHFR 677C>T*** | | | | | |
| *MTRR 66AA + MTHFR 677CC* | 57 | 27 | 50 | 0.810 | 1 |
| *MTRR 66AA + MTHFR 677TT* | 23 | 12 | 51 |  | 1.046(0.528-2.072) |
| *MTRR 66GG + MTHFR 677CC* | 3 | 1 | 84b |  | 0.427(0.057-3.202) |
| *MTRR 66GG + MTHFR 677TT* | 3 | 2 | 35 |  | 1.349(0.319-5.700) |
| ***MTRR 66A>G and MTR 2756A>G*** | | | | | |
| *MTRR 66AA + MTR 2756AA* | 105 | 54 |  | 0.057 | 1 |
| *MTRR 66AA + MTR 2756GG* | 1 | 0 |  |  | 0 |
| *MTRR 66GG + MTR 2756AA* | 6 | 0 |  |  | 0.041(0.09-2.983) |
| *MTRR 66GG + MTR 2756GG* |  |  |  |  |  |
| ***MTRR 66A>G* and *TS*5-UTR 2R>3R** | | | | | |
| *MTRR 66AA + TS*5-UTR 2R2R | 8 | 5 | 39 | 0.449 | 1 |
| *MTRR 66AA + TS*5-UTR 3R3R | 77 | 34 | 52 |  | 0.713(0.276-1.838) |
| *MTRR 66GG + TS*5-UTR 2R2R | 1 | 1 | 20 |  | 2.121(0.246-18.31) |
| *MTRR 66GG + TS5*-UTR 3R3R | 9 | 3 | 76b |  | 0.427(0.101-1.816) |
| ***MTRR 66A>G and TS*3-UTR I>D** | | | | | |
| *MTRR 66AA + TS*3-UTRII | 13 | 6 |  | 0.288 | 1 |
| *MTRR 66AA + TS*3-UTR DD | 55 | 23 |  |  | 0.913(0.371-2.248) |
| *MTRR 66GG + TS*3-UTR II | 1 | 1 |  |  | 2.54(0.303-21.301) |
| *MTRR 66GG + TS*3-UTR DD | 4 | 0 |  |  | 0 |
| ***MTHFR 1298A>C and MTHFR 677C>T*** | | | | | |
| *MTHFR 1298AA + MTHFR677CC* | 50 | 21 | 62 | 0630 | 1 |
| *MTHFR 1298AA + MTHFR677TT* | 41 | 17 | 55 |  | 1.009(0.532-1.914) |
| *MTHFR 1298CC + MTHFR677CC* | 8 | 2 | 69b |  | 0.508(0.119-2.169) |
| *MTHFR 1298CC + MTHFR677TT* |  |  |  |  |  |
| ***MTHFR 1298A>C and MTR 2756A>G*** | | | | | |
| *MTHFR 1298AA + MTR2756AA* | 132 | 58 |  | 0.413 | 1 |
| *MTHFR 1298AA + MTR2756GG* | 1 | 0 |  |  | 0 |
| *MTHFR 1298CC + MTR2756AA* | 8 | 2 |  |  | 0.452(0.110-1.851) |
| *MTHFR 1298CC + MTR2756GG* |  |  |  |  |  |
| ***MTHFR 1298A>C and TS5-UTR 2R>3R*** | | | | | |
| *MTHFR 1298AA + TS*5-UTR 2R2R | 8 | 4 |  | 0.162 | 1 |
| *MTHFR 1298AA+ TS*5-UTR 3R3R | 101 | 41 |  |  | 0.673(.240-1.891) |
| *MTHFR 1298CC + TS*5-UTR 2R2R | 1 | 1 |  |  | 2.700(0.29-24.454) |
| *MTHFR 1298CC+ TS*5-UTR 3R3R | 4 | 0 |  |  | 0 |
| ***MTHFR 1298A>C and TS*3-UTR I>D** | | | | | |
| *MTHFR 1298AA+ TS*3-UTR II | 16 | 9 |  | 0.208 | 1 |
| *MTHFR 1298AA + TS*3-UTR DD | 74 | 30 |  |  | 0.821(0.376-1.791) |
| *MTHFR 1298CC + TS*3-UTR II | 1 | 1 |  |  | 2.784(0.343-2.572) |
| *MTHFR 1298CC+ TS*3-UTR DD | 4 | 0 |  |  |  |
| ***MTHFR 677C>T and MTR2756A>G*** | | | | | |
| *MTHFR 677CC+ MTR2756AA* | 73 | 2 |  | 0.853 | 1 |
| *MTHFR 677CC+ MTR2756GG* | 1 | 0 |  |  | 0 |
| *MTHFR 677TT+ MTR2756AA* | 33 | 13 |  |  | 0.973(0.505-1.875) |
| *MTHFR 677TT+ MTR2756GG* | 1 | 0 |  |  | 0 |
| ***MTHFR 677C>T and TS*5-UTR 2R>3R** | | | | | |
| *MTHFR 677CC + TS*5-UTR 2R2R | 5 | 4 | 39 | 0.121 | 1 |
| *MTHFR 677CC+ TS*5-UTR 3R3R | 53 | 16 | 76b |  | 0.312(0.102-0.948) |
| *MTHFR 677TT+ TS*5-UTR 2R2R | 4 | 2 | 20 |  | 0.747(0.126-4.086) |
| *MTHFR 677TT + TS*5-UTR 3R3R | 23 | 8 | 61b |  | 0.323(0.095-1.099) |
| ***MTHFR 677C>T and TS*3-UTR I>D** | | | | | |
| *MTHFR 677CC+ TS*3-UTRII | 8 | 4 | 46 | 0.986 | 1 |
| *MTHFR 677CC + TS*3-UTR DD | 81 | 32 | 75b |  | 0.834(0.294-2.367) |
| *MTHFR 677TT + TS*3-UTR II | 2 | 1 | 20 |  | 0.851(0.095-7.660) |
| *MTHFR 677TT + TS*3-UTR DD | 19 | 8 | 55 |  | 0.789(0.240-2.655) |
| ***MTR 2756A>G and TS*5-UTR 2R>3R** | | | | | |
| *MTR 2756AA + TS*5-UTR 2R2R | 11 | 7 |  | 0.194 | 1 |
| *MTR 2756AA+ TS*5-UTR 3R3R | 107 | 39 |  |  | 0.504(0.224-1.134) |
| *MTR 2756 GG + TS*5-UTR 2R2R | 1 | 0 |  |  | 0 |
| *MTR 2756 GG + TS*5-UTR 3R3R |  |  |  |  |  |
| ***MTR 2756A> G and TS*3-UTR I>D** | | | | | |
| *MTR 2756AA + TS*3-UTRII | 15 | 6 |  | 0.665 | 1 |
| *MTR 2756AA + TS*3-UTR DD | 79 | 30 |  |  | 0.939(0.390-2.259) |
| *MTR 2756 GG + TS* 3-UTR II | 2 | 0 |  |  | 0 |
| *MTR 2756 GG + TS* 3-UTR DD |  |  |  |  |  |
| ***TS*3-UTR I>D and *TS* 5-UTR 2R>3R** | | | | | |
| *TS*3-UTR II+ *TS*5-UTR 2R2R | 5 | 5 | 18 | <0.001 | 1 |
| *TS*3-UTR II+ *TS*5-UTR 3R3R | 4 | 2 | 46 |  | 0.336(0.064-1.765) |
| *TS*3-UTR DD+ *TS*5-UTR 2R2R | 1 | 1 |  |  | 2.665(0.294-24.16) |
| *TS*3-UTR DD+ *TS*5-UTR 3R3R | 87 | 29 | 74b |  | 0.212(0.080-0.563) |

^a^Adjusted for age and sex.

^b^Mean survival time was provided when MST could not be calculated.

**Supplementary table S2**. The effects of gene-gene interactions in heterozygous model on the survival of gastric cancer patients receiving 5-FU–based chemotherapy

| **combined genotypes** | **patient** | **death** | **MST** | **P** | **HR (95%CI)^a^** |
| --- | --- | --- | --- | --- | --- |
| ***MTRR 66A>G and MTHFR 1298A>C*** | | | | | |
| *MTRR 66AA + MTHFR 1298AA* | 95 | 49 | 46 | 0.307 | 1 |
| *MTRR 66AA + MTHFR 1298CA* | 35 | 16 | 51b |  | 0.864(0.491-1.521) |
| *MTRR 66GA + MTHFR 1298AA* | 68 | 24 | 72b |  | 0.627(0.384-1.023) |
| *MTRR 66GA + MTHFR 1298CA* | 27 | 12 | 65b |  | 0.856(0.455-1.610) |
| ***MTRR 66A>G and MTHFR 677C>T*** | | | | | |
| *MTRR 66AA + MTHFR 677CC* | 57 | 27 | 50 | 0.292 | 1 |
| *MTRR 66AA + MTHFR 677TC* | 54 | 29 | 36 |  | 1.199(0.710-2.026) |
| *MTRR 66GA + MTHFR 677CC* | 31 | 10 | 77b |  | 0.604(0.292-1.250) |
| *MTRR 66GA + MTHFR 677TC* | 53 | 23 | 59b |  | 0.933(0.535-1.628) |
| ***MTRR 66A>G and MTR 2756A>G*** | | | | | |
| *MTRR 66AA + MTR 2756AA* | 110 | 55 | 50 | 0.427 | 1 |
| *MTRR 66AA + MTR 2756GA* | 18 | 7 | 52 |  | 0.693(0.315-1.523) |
| *MTRR 66GA + MTR 2756AA* | 76 | 28 | 71b |  | 0.703(0.446-1.110) |
| *MTRR 66GA + MTR 2756GA* | 16 | 7 | 62b |  | 0.824(0.374-1.811) |
| ***MTRR 66A>G and TS*5-UTR 2R>3R** | | | | | |
| *MTRR 66AA + TS*5-UTR 2R2R | 8 | 5 | 39 | 0.126 | 1 |
| *MTRR 66AA + TS*5-UTR 2R3R | 42 | 24 | 20 |  | 1.201(0.457-3.158) |
| *MTRR 66GA + TS*5-UTR 2R2R | 5 | 4 | 12 |  | 2.914(0.774-10.97) |
| *MTRR 66GA + TS*5-UTR 2R3R | 31 | 13 | 61b |  | 0.808(0.288-2.272) |
| ***MTRR 66A>G and TS*3-UTR I>D** | | | | | |
| *MTRR 66AA + TS*3-UTRII | 13 | 6 | 46 | 0.288 | 1 |
| *MTRR 66AA + TS*3-UTR DI | 64 | 38 | 27 |  | 1.448(0.612-3.427) |
| *MTRR 66GA + TS*3-UTR II | 8 | 4 | 18 |  | 1.113(0.313-3.955) |
| *MTRR 66GA + TS*3-UTR DI | 44 | 16 | 66b |  | 0.839(0.328-2.146) |
| ***MTHFR 1298A>C and MTHFR 677C>T*** | | | | | |
| *MTHFR 1298AA + MTHFR677CC* | 50 | 21 | 62 | 0.881 | 1 |
| *MTHFR 1298AA + MTHFR677TC* | 83 | 39 | 43 |  | 1.242(0.731-2.112) |
| *MTHFR 1298CA + MTHFR677CC* | 32 | 14 | 72b |  | 1.146(0.583-2.255) |
| *MTHFR 1298CA + MTHFR677TC* | 31 | 14 | 60b |  | 1.111(0.565-2.186) |
| ***MTHFR 1298A>C and MTR 2756A>G*** | | | | | |
| *MTHFR 1298AA + MTR2756AA* | 132 | 58 | 55 | 0.950 | 1 |
| *MTHFR 1298AA + MTR2756GA* | 28 | 12 | 63 |  | 0.904(0.485-1.685) |
| *MTHFR 1298CA + MTR2756AA* | 47 | 22 | 69 |  | 1.109(0.679-1.813) |
| *MTHFR 1298CA + MTR2756GA* | 11 | 5 | 50b |  | 1.020(0.409-2.546) |
| ***MTHFR 1298A>C and TS*5-UTR 2R>3R** | | | | | |
| *MTHFR 1298AA + TS*5-UTR 2R2R | 8 | 4 | 39 | 0.413 | 1 |
| *MTHFR 1298AA + TS*5-UTR 2R3R | 55 | 27 | 51 |  | 0.953(0.333-2.728) |
| *MTHFR 1298CA + TS*5-UTR 2R2R | 5 | 5 | 23 |  | 2.091(0.561-7.795) |
| *MTHFR 1298CA + TS*5-UTR 2R3R | 17 | 8 | 67b |  | 0.998(0.299-3.326) |
| ***MTHFR 1298A>C and TS*3-UTR I>D** | | | | | |
| *MTHFR 1298AA + TS*3-UTRII | 16 | 8 | 46 | 0.930 | 1 |
| *MTHFR 1298AA + TS*3-UTR DI | 82 | 39 | 50 |  | 0.967(0.452-2.069) |
| *MTHFR 1298CA + TS*3-UTR II | 5 | 2 | 38b |  | 0.723(0.153-3.409) |
| *MTHFR 1298CA + TS*3-UTR DI | 31 | 16 | 39 |  | 1.112(0.476-2.600) |
| ***MTHFR 677C>T and MTR 2756A>G*** | | | | | |
| *MTHFR 677CC+ MTR2756AA* | 73 | 29 | 73b | 0.561 | 1 |
| *MTHFR 677CC+ MTR2756GA* | 11 | 5 | 61b |  | 1.181(0.456-3.056) |
| *MTHFR 677TC+ MTR2756AA* | 82 | 40 | 37 |  | 1.381(0.856-2.229) |
| *MTHFR 677TC+ MTR2756GA* | 20 | 8 | 65b |  | 1.00(0.457-2.190) |
| ***MTHFR 677C>T and TS*5-UTR 2R>3R** | | | | | |
| *MTHFR 677CC + TS*5-UTR 2R2R | 5 | 4 | 39 | 0.483 | 1 |
| *MTHFR 677CC+ TS*5-UTR 2R3R | 29 | 14 | 68b |  | 0.726(0.239-2.207) |
| *MTHFR 677TC+ TS*5-UTR 2R2R | 5 | 4 | 18 |  | 1.614(0.401-6.491) |
| *MTHFR 677TC + TS*5-UTR 2R3R | 34 | 16 | 58b |  | 0.755(0.252-2.261) |
| ***MTHFR 677C>T and TS*3-UTR I>D** | | | | | |
| *MTHFR 677CC+ TS*3-UTRII | 8 | 4 | 46 | 0.975 | 1 |
| *MTHFR 677CC + TS*3-UTR DI | 47 | 24 | 50 |  | 1.218(0.422-3.516) |
| *MTHFR 677TC + TS*3-UTR II | 12 | 6 | 18 |  | 1.342(0.378-4.770) |
| *MTHFR 677TC + TS*3-UTR DI | 48 | 24 | 37 |  | 1.208(0.418-3.488) |
| ***MTR 2756A>G and TS*5-UTR 2R>3R** | | | | | |
| *MTR 2756AA + TS*5-UTR 2R2R | 11 | 7 | 39 | 0.245 | 1 |
| *MTR 2756AA + TS*5-UTR 2R3R | 61 | 30 | 51 |  | 0.808(0.354-1.844) |
| *MTR 2756GA + TS*5-UTR 2R2R | 3 | 3 | 18 |  | 2.475(0.633-9.672) |
| *MTR 2756GA + TS*5-UTR 2R3R | 9 | 4 | 61b |  | 0.706(0.205-2.428) |
| ***MTR 2756A> G and TS*3-UTR I>D** | | | | | |
| *MTR 2756AA + TS*3-UTRII | 15 | 6 | 53b | 0.556 | 1 |
| *MTR 2756AA + TS*3-UTR DI | 91 | 45 | 39 |  | 1.358(0.579-3.183) |
| *MTR 2756GA + TS*3-UTR II | 7 | 5 | 20 |  | 1.925(0.587-6.311) |
| *MTR 2756GA + TS*3-UTR DI | 14 | 5 | 68b |  | 0.886(0.270-2.905) |
| ***TS*3-UTR I>D and *TS*5-UTR 2R>3R** | | | | | |
| *TS*3-UTR II+ *TS*5-UTR 2R2R | 5 | 5 | 18 | 0.111 | 1 |
| *TS*3-UTR II+ *TS*5-UTR 2R3R | 12 | 3 | 77b |  | 0.192(0.046-0.806) |
| *TS*3-UTR DD+ *TS*5-UTR 2R2R | 8 | 4 | 39 |  | 0.395(0.105-1.482) |
| *TS*3-UTR DD+ *TS*5-UTR 2R3R | 52 | 27 | 27 |  | 0.493(0.189-1.286) |

^a^Adjusted for age and sex.

^b^Mean survival time was provided when MST could not be calculated.

**Supplementary Table S3.** The effects of gene-gene interactions in dominant model on the survival of gastric cancer patients receiving 5-FU–based chemotherapy

| **combined genotypes** | **patient** | **death** | **MST** | ***P*** | **HR (95%CI)^a^** |
| --- | --- | --- | --- | --- | --- |
| ***MTRR 66A>G and MTHFR 1298A>C*** | | | | | |
| *MTRR 66AA + MTHFR 1298AA* | 95 | 49 | 46 | 0.187 | 1 |
| *MTRR66AA+MTHFR1298CC+CA* | 39 | 18 | 51b |  | 0.875(0.510-1.502) |
| *MTRR 66GG+GA + MTHFR 1298AA* | 78 | 28 | 72b |  | 0.632(0.397-1.006) |
| *MTRR66GG+GA+MTHFR1298CC+CA* | 33 | 12 | 81b |  | 0.638(0.339-1.200) |
| ***MTRR 66A>G and MTHFR 677C>T*** | | | | | |
| *MTRR 66AA + MTHFR 677CC* | 57 | 27 | 50 | 0.133 | 1 |
| *MTRR 66AA + MTHFR 677TT+TC* | 77 | 41 | 43 |  | 1.146(0.705-1.864) |
| *MTRR 66GG+GA + MTHFR 677CC* | 34 | 11 | 85b |  | 0.588(0.291-1.187) |
| *MTRR 66GG+GA + MTHFR 677TT+TC* | 77 | 29 | 64b |  | 0.761(0.451-1.287) |
| ***MTRR 66A>G and MTR 2756A>G*** | | | | | |
| *MTRR 66AA + MTR 2756AA* | 105 | 54 | 43 | 0.149 | 1 |
| *MTRR 66AA + MTR 2756GG+GA* | 19 | 7 | 65b |  | 0.631(0.287-1.388) |
| *MTRR 66GG+GA + MTR 2756AA* | 82 | 28 | 81b |  | 0.607(0.384-0.959) |
| *MTRR 66GG+GA + MTR 2756GG+GA* | 21 | 10 | 60b |  | 0.871(0.443-1.713) |
| ***MTRR 66A>G and TS*5-UTR 2R>3R** | | | | | |
| *MTRR 66AA + TS*5-UTR 2R2R | 8 | 5 | 39 | 0.002 | 1 |
| *MTRR 66AA + TS*5-UTR 2R2R+2R3R | 119 | 58 | 51 |  | 0.905(0.362-2.259) |
| *MTRR 66GG+GA + TS*5-UTR 2R2R | 6 | 5 | 12 |  | 2.648(0.763-9.192) |
| *MTRR66GG+GA+TS*5-UTR2R2R+2R3R | 97 | 32 | 83b |  | 0.540(0.210-1.389) |
| ***MTRR 66A>G and TS* 3-UTRI>D** | | | | | |
| *MTRR 66AA + TS*3-UTR II | 13 | 6 | 46 | 0.101 | 1 |
| *MTRR66AA + TS*3-UTR II+DI | 119 | 61 | 50 |  | 1.204(0.520-2.786) |
| *MTRR 66GG+GA + TS*3-UTR II | 9 | 5 | 20 |  | 1.262(0.385-4.139) |
| *MTRR 66GG+GA + TS*3-UTR II+DI | 99 | 34 | 82b |  | 0.720(0.302-1.717) |
| ***MTHFR 1298A>C and MTHFR 677C>T*** | | | | | |
| *MTHFR 1298AA + MTHFR677CC* | 50 | 21 | 62 | 0.926 | 1 |
| *MTHFR 1298AA + MTHFR677TT+TC* | 124 | 56 | 55 |  | 1.155(0.700-1908) |
| *MTHFR 1298CC+CA + MTHFR677CC* | 40 | 16 | 76b |  | 1.002(0.523-1.921) |
| *MTHFR1298CC+CA+MTHFR677TT+TC* | 31 | 14 | 60b |  | 1.105(0.562-2.174) |
| ***MTHFR 1298A>C and MTR 2756A>G*** | | | | | |
| *MTHFR 1298AA + MTR2756AA* | 132 | 58 | 55 | 0.977 | 1 |
| *MTHFR 1298AA + MTR2756GG+GA* | 29 | 12 | 64b |  | 0.873(0.468-1.626) |
| *MTHFR 1298CC+CA + MTR2756AA* | 55 | 24 | 73b |  | 0.994(0.618-1.601) |
| *MTHFR1298CC+CA+MTR2756GG+GA* | 12 | 5 | 52b |  | 0.937(0.375-2.337) |
| ***MTHFR 1298A>C and TS* 5-UTR 2R>3R** | | | | | |
| *MTHFR 1298AA + TS*5-UTR 2R2R | 8 | 4 | 39 | 0.027 | 1 |
| *MTHFR1298AA+TS*5-UTR 2R2R+2R3R | 156 | 68 | 64b |  | 0.791(0.288-2.171) |
| *MTHFR 1298CC+CA+ TS*5-UTR 2R2R | 6 | 6 | 18 |  | 2.217(0.625-7.863) |
| *MTHFR1298CC+CA+TS*5-UTR2R2R+2R3R | 61 | 21 | 882b |  | 0.590(0.202-1.723) |
| ***MTHFR 1298A>C and TS* 3-UTRI>D** | | | | | |
| *MTHFR 1298AA + TS*3-UTR II | 16 | 8 | 46 | 0.897 | 1 |
| *MTHFR 1298AA+ TS*3-UTR DD+DI | 156 | 69 | 62 |  | 0.890(0.428-1.850) |
| *MTHFR 1298CC+CA + TS*3-UTR II | 6 | 3 | 18 |  | 0.985(0.261-3.716) |
| *MTHFR 1298CC+CA + TS*3-UTR DD+DI | 63 | 25 | 77b |  | 0.773(0.349-1.714) |
| ***MTHFR 677C>T and MTR 2756A>G*** | | | | | |
| *MTHFR 677CC+ MTR2756AA* | 73 | 29 | 73b | 0.784 | 1 |
| *MTHFR 677CC+ MTR2756GG+GA* | 12 | 5 | 64b |  | 1.049(0.405-2.713) |
| *MTHFR 677TT+TC + MTR2756AA* | 115 | 53 | 55 |  | 1.250(0.795-1.966) |
| *MTHFR 677TT+TC + MTR2756GG+GA* | 28 | 12 | 63b |  | 1.058(0.540-2.075) |
| ***MTHFR 677C>T and TS* 5-UTR 2R>3R** | | | | | |
| *MTHFR 677CC + TS*5-UTR 2R2R | 5 | 4 | 39 | 0.124 | 1 |
| *MTHFR 677CC+ TS*5-UTR 2R2R+2R3R | 82 | 30 | 79b |  | 0.466(0.164-1.324) |
| *MTHFR 677TT+TC+ TS*5-UTR 2R2R | 9 | 6 | 20 |  | 1.153(0.325-4.091) |
| *MTHFR677TT+TC+TS*5-UTR2R2R+2R3R | 135 | 59 | 61b |  | 0.578(0.210-1.595) |
| ***MTHFR 677C>T and TS* 3-UTR I>D** | | | | | |
| *MTHFR 677CC+ TS*3-UTRII | 8 | 4 | 46 | 0.810 | 1 |
| *MTHFR 677CC + TS*3-UTR DD+DI | 81 | 32 | 75b |  | 0.870(0.307-2.462) |
| *MTHFR 677TT+TC + TS*3-UTR II | 14 | 7 | 20 |  | 1.214(0.355-4.153) |
| *MTHFR 677TT+TC + TS*3-UTR DD+DI | 137 | 62 | 55 |  | 1.035(0.376-2.846) |
| ***MTR 2756A>G and TS*5-UTR 2R>3R** | | | | | |
| *MTR 2756AA + TS*5-UTR 2R2R | 11 | 7 | 39 | 0.019 | 1 |
| *MTR 2756AA + TS*5-UTR 2R2R+2R3R | 168 | 69 | 73b |  | 0.603(0.27-1.315) |
| *MTR 2756GG+GA + TS*5-UTR 2R2R | 3 | 3 | 18 |  | 2.558(0.657-9.955) |
| *MTR2756GG+GA+TS*5-UTR2R2R+2R3R | 34 | 12 | 70b |  | 0.458(0.180-1.169) |
| **MTR 2756A>G and TS3-UTR I>D** | | | | | |
| MTR 2756 AA + TS3-UTRII | 15 | 6 | 53b | 0.378 | 1 |
| MTR 2756 AA + TS3-UTR DD+DI | 170 | 75 | 62 |  | 1.151(0.501-2.645) |
| MTR 2756GG+GA + TS3-UTR II | 7 | 5 | 20 |  | 1.928(0.588-6.322) |
| MTR 2756GG+GA + TS3-UTR DD+DI | 33 | 11 | 71b |  | 0.788(0.291-2.132) |
| ***TS*3-UTR I>D and *TS*5-UTR 2R>3R** | | | | | |
| *TS*3-UTR II+ *TS*5-UTR 2R2R | 5 | 5 | 18 | 0.046 | 1 |
| *TS*3-UTR II+ *TS*5-UTR 2R3R+3R3R | 16 | 5 | 72b |  | 0.223(0.064-0.776) |
| *TS*3-UTR DD+DI + *TS*5-UTR 2R2R | 9 | 5 | 39 |  | 0.481(0.139-1.666) |
| *TS*3-UTRDD+DI+ *TS*5-UTR 2R3R+3R3R | 202 | 85 | 73b |  | 0.332(0.134-0.822) |

^a^Adjusted for age and sex.

^b^Mean survival time was provided when MST could not be calculated.

**Supplementary Table S4.** Primers for the genotyping assays of *MTRR, MTHFR, MTR and TYMS*

| **Genotypes** | **Forward primer** | **Reverse primer** | **Extension primer** |
| --- | --- | --- | --- |
| ***MTRR* rs1801394 66A>G** | ACACAGCAGGGACAGGCAAAG | GCAGAAAATCCATGTACCACAGC | TTTTTTTTTTTTTAAGGCCATCGCAGAAGAAAT |
| ***MTHFR* rs1801131 1298 A >C** | AAGGAGGAGCTGCTGAAGATGTG | TGGTTCTCCCGAGAGGTAAAGAACA | TTTTTTTTTTTTTTTTTTTTTTTTTTTTTTGGTAAAGAACRAAGACTTCAAAGACACKT |
| ***MTHFR* rs1801133 677 C> T** | TGAGGCTGACCTGAAGCACTTG | CAAAGAAAAGCTGCGTGATGATGA | TTTTTTTTGCTGCGTGATGATGAAATCG |
| ***MTR* rs1805087 2756 A >G** | AAGGATGAATACTTTGAGGAAATCATGG | CTGTTTCTACCACTTACCTTGAGAGACTCAT | TTTTTTTTTTTTTTTTTTCATGGAAGAATATGAAGATATTAGACAGG |
| ***TYMS* 5-UTR 2R > 3R** | CGGAAGGGGTCCTGCCACC | GAGCCGGCCACAGGCATGG |  |
| ***TYMS* 3-UTR 6bp ins>del** | GGAGCTGAGTAACACCATCGATCA | GCGTGGACGAATGCAGAACA |  |
